# Dataset on the total phenolic contents and total anti-oxidants capacity in commercially available whiskey

**DOI:** 10.1016/j.dib.2022.108608

**Published:** 2022-10-08

**Authors:** Ali J. Othman, Sarah G. Saker

**Affiliations:** a Department of Commodity Research and Commodity Expertise, Plekhanov Russian University of Economics, Moscow, Russia; b Tishreen University, Department of food analysis, Latakia, Syria Arab Republic

**Keywords:** Whiskey, Total phenolic content, Total anti-oxidants capacity, Aged whiskey

## Abstract

Data in this article are acquired from 46 naturally distilled whiskey. Whiskey samples were produced in the United Kingdom, United States, Ireland, Scotland, and Canada. Samples differ with their type of distillery including (Blended, finest blended, Malted, single Malted, rye, straight Rye, special blended, and special reserved), years of aging, and ethanol percentages. The contents of beneficial bioactive components including total phenolic contents and total anti-oxidants activity and the correlation between them were addressed.

**Specifications Table**

**Table:** 

Subject	Food science
Specific subject area	Total phenolic content and total anti-oxidant capacity in aged Whiskey
Type of data	Table
How the data were acquired	Coulometric analyzer EXPERT-006 (Econix-Expert, Russia) Spectrophotometer (Shimadzu UV 2401pc, Japan) Sodium carbonate, powder, ≥99.5%, ACS reagent (Sigma-Aldrich, st. louis, USA) Ascorbic acid 99% (Sigma-Aldrich, st. louis, USA) Folin-Ciocalteu's phenol reagent (Sigma-Aldrich, st. louis, USA) Gallic acid reference substance ≥98.0% (Sigma-Aldrich, st. louis, USA) Potassium bromide ACS Reagent (Supelco, Pennsylvania, USA) Sulfuric acid 0.5 mol. L^−1^ (1 N), Ph. Eur., reag. USP (Supelco, Pennsylvania, USA)
Data format	Raw data provided in Microsoft Excel Worksheet Data link to a repository website:
Description of data collection	46 samples of naturally distilled whiskey were purchased from the supermarket (Perekrostok, Moscow, Russia). Whiskey samples were produced in the United Kingdom, United States, Ireland, Scotland, and Canada. Samples differ with their type of distillery including (Blended, finest blended, Malted, single Malted, rye, straight Rye, special blended, and special reserved), years of aging, and ethanol percentages. The data on total phenolic content were obtained by the colorimetric method of Folin–Ciocalteu using gallic acid as a standard [1]. The data on total antioxidants capacity was obtained by coulometric analysis method using electrogenerated bromine radicals as described by Lapin A. [2,3] with modifications. Microsoft Excel Worksheet containing mean values of total phenolic contents and total anti-oxidants capacity, years of aging, type of distillery, and alcohol percentages of 46 sorts of Whiskey
Data source location	Institution: Plekhanov Russian University of Economics City/Town/Region: Moscow Country: Russian Federation Whiskey samples were produced in Scotland, Ireland, Canada, The United States, and United Kingdom.
Data accessibility	With the article Repository name: https://data.4tu.nl/ Data identification number: 10.4121/20337516

## Value of the Data

• These data can add an additional information regarding the content of total phenolic content and anti-oxidant capacity of 46 common whisky commodity
• This dataset is necessary as a qualification approach to the systematic identification of whiskey products
• The method of determination of total anti-oxidants capacity described in this manuscript is precise, relevant, and short time consuming that can be used by oenologists and whiskey researchers

## 1 Data Description

This dataset contains analyzed data obtained by galvanostatic coulometric analyzer and spectrophotometer from freshly opened whiskey bottles obtained from the supermarket. Whiskey samples were produced in the United Kingdom, United States, Ireland, Scotland, and Canada. Samples differ with their type of distillery including (Blended, finest blended, Malted, single Malted, rye, straight Rye, special blended, and special reserved), years of aging, and ethanol percentages. Dataset provided about total phenolics content and total anti-oxidants capacity is displayed in Table 1. Data on years of distillation was obtained from the labelling of each bottle. Correlations between total phenolic contents, total anti-oxidants capacity, and distillation years are shown in Table 2.

**Table 1:** Distillery type, percentage of alcohol, distillation years, total phenolic contents, and total anti-oxidant capacity of 46 whiskey samples.

No	Name of Whiskey	Distillery type	Alcohol, %	Aged	Total phenolic contents, (mg GAE/mL)	Total Anti-oxidant capacity, (mg AA/100mL)
1	JAMESON Crested x Eight Degrees Brewing	Blended	43	*	4.24 ± 0.01	32.92 ± 0.88
2	JAMESON IRISH WHISKEY	Blended	40	4	4.26 ± 0.07	21.6 ± 0.09
3	JAMESON Black Barrel	Blended	40	12	4.58 ± 0.08	28.2 ± 0.60
4	JIM BEAM BOURBON WHISKEY	Blended	40	4	3.27 ± 0.03	47.3 ± 0.87
5	JIM BEAM BOURBON WHISKEY - Honey	Blended	35	4	3.09 ± 0.14	66.4 ± 0.50
6	JIM BEAM BOURBON WHISKEY Black Extra-aged	Blended	43	8	3.62 ± 0.06	60.0 ± 0.03
7	JACK DANIEL'S Straight Rye Tennessee Whiskey	Rye	45	5	4.38 ± 0.11	59.5 ± 0.58
8	JACK DANIEL'S Old No. 7	Blended	40	5	4.43 ± 0.08	48.9 ± 0.15
9	JACK DANIEL'S Tennessee Honey Whiskey	Blended	35	5	7.59 ± 0.14	76.4 ± 0.48
10	JACK DANIEL'S GENTLEMAN JACK Whiskey	Double mellowed	40	10	6.55 ± 0.27	70.8 ± 0.53
11	Ballantine's finest blended Scotch whisky	Blended	40	18	5.64 ± 0.02	78.6 ± 0.41
12	Ballantine's 17 Year Old Whisky	Blended	40	17	5.55 ± 0.14	82.1 ± 0.68
13	Ballantine's 12 Year Old Whisky	Blended	40	12	4.60 ± 0.21	79.4 ± 0.24
14	Ballantine's 30 Year Old Whisky	Blended	40	30	6.45 ± 0.18	83.0 ± 0.40
15	Ballantine's Glenburgie 15 Year Old Whisky	Blended	40	15	5.54 ± 0.42	71.3 ± 0.29
16	Buchanan Scotch Whisky	Finest blended	40	12	12.16 ± 0.19	33.26 ± 0.60
17	Buchanan Scotch Whisky (De luxe)	Blended	40	12	6.93 ± 0.14	73.8 ± 0.52
18	Buchanan Scotch Whisky	Single Malted	40	15	15.186 ± 0.12	51.98 ± 0.72
19	Buchanan Scotch Whisky (special reserved)	Special blended and Special reserved	40	18	7.80 ± 0.11	64.7 ± 0.90
20	Buchanan Scotch Whisky	Blended	40	21	21.21 ± 0.00	73.21 ± 0.40
21	Loch Lomond Single Malt Scotch Whisky Highland	Single Malted	40	12	7.39 ± 0.09	77.7 ± 0.54
22	Loch Lomond Single Malt Scotch Whisky Highland	Single Malted	40	18	7.64 ± 0.09	53.9 ± 0.42
23	Loch Lomond Single Malt Scotch Whisky Highland	Single Malted	40	21	7.08 ± 0.04	63.6 ± 0.41
24	Johnnie Walker Green Label blended malt Scotch whisky	Malted	43	15	6.48 ± 0.25	54.7 ± 0.01
25	Johnnie Walker Blue Label blended Scotch whisky	Malted	43	28	7.49 ± 0.02	71.4 ± 0.26
26	Johnnie Walker Red Label blended Scotch whisky	Blended	40	12	7.12 ± 0.10	32.6 ± 0.12
27	Glenfiddich Special Reserve single malt Scotch whisky	Single Malted	43	12	5.59 ± 0.06	33.9 ± 0.34
28	Glenfiddich Special Reserve single malt Scotch whisky	Single Malted	40	12	5.81 ± 0.06	36.8 ± 0.03
29	Glenfiddich Special Reserve single malt Scotch whisky	Single Malted	40	15	6.50 ± 0.05	52.9 ± 0.12
30	Chivas Regal blended Scotch whisky	Blended	40	12	6.69 ± 0.20	40.2 ± 0.70
31	Chivas Regal blended Scotch whisky	Blended	40	18	7.11 ± 0.23	57.5 ± 0.48
32	Clontarf Irish whiskey	Blended	40	*	3.26 ± 0.17	21.76 ± 0.16
33	Clontarf Irish whiskey reserve	Blended	40	*	6.81 ± 0.09	22.6 ± 0.17
34	Clontarf 1014 Black Label Classic Blend	Single Malted	40	4	4.40 ± 0.08	19.9 ± 0.32
35	Clontarf 1014 Irish whiskey	Single Malted	40	3	3.48 ± 0.05	19.1 ± 0.11
36	Clan Denny blended malt Scotch whisky from speyside	Malted	46.7	3	2.65 ± 0.02	17.2 ± 0.33
37	Glenkinchie malt lowland Scotch whisky	Malted	43	10	3.62 ± 0.06	49.9 ± 0.11
38	Glenfiddich Special Reserve single malt Scotch whisky	Single Malted	40	18	7.71 ± 0.07	78.9 ± 0.17
39	Dewar's	Blended	40	12	4.25 ± 0.05	35.5 ± 0.33
40	GlenClyde	Blended	40	12	4.33 ± 0.08	30.1 ± 0.14
41	Crown Royal Canadian Whisky	Blended	40	13	6.14 ± 0.10	36.7 ± 0.15
42	The Girvan Patent Still	Blended	42	25	7.60 ± 0.30	75.93 ± 0.19
43	The Girvan Patent Still No4	Blended	42	10	5.69 ± 0.08	45.65 ± 0.07
44	Grant's Triple Wood	Blended	40	3	2.38 ± 0.12	19.23 ± 0.13
45	Monkey Shoulder	Blended	40	*	4.71 ± 0.05	30.81 ± 1.33
46	Glenfiddich	Single malted	40	12	5.64 ± 0.34	19.23 ± 0.14

⁎ no-age-statement whisky

**Table 2:** Correlations between total phenolic contents, total anti-oxidants capacity, and distillation years.

	Total Phenolic Contents	Total Anti-oxidants Capacity	Aged
Total Phenolic Contents		.516⁎⁎	.707⁎⁎
Total Anti-oxidants Capacity	.516⁎⁎		.568⁎⁎
Aged	.707⁎⁎	.568⁎⁎	

⁎⁎ Correlation is significant at the 0.01 level (2-tailed).

## 2 Experimental Design, Materials and Methods

### 2.1 Total phenols content

This method is based on the oxidation of phenolic compounds with a Folin-Chocalteu reagent. The reagent is a mixture of phosphoric-tungsten H3PW12O40 and phosphoric-molybdenum H3PMo12O40 acids, which are reduced by oxidation due to the existence of phenols into a mixture of oxides including blue tungsten (W8O23) and molybdenum (MO8O23). Blue staining makes it possible to measure the maximum absorption of the solution. It is proportional to the content of phenolic compounds.

Sample preparation comprises the preparation of a reaction mixture including 30 ml of whiskey, 2220 mL distilled water, 150 mL of Folin-Chocalteu reagent, 600 ml of a 20% solution of sodium carbonate (Na2CO3).

A control sample was also prepared, where distilled water was used instead of whiskey. Thus, the composition of the control sample was as follows: 2250 µl distilled water, 150 µl of Folin-Chocalteu reagent, 600 µl of a 20% solution of sodium carbonate (Na2CO3).

Further, the obtained solutions were kept for 90 min at room conditions until the complete completion of the oxidation reaction of phenolic compounds, when the optical density practically did not change during subsequent measurements, which made it possible to reduce the error several times.

Optical density measurements were carried out at a wavelength of 750 nm using (Shimadzu UV 2401pc, Japan) spectrophotometer. Measurements were carried out in 1 cm thick cuvettes. Total phenolic content was expressed as mg gallic acid equivalents (GAE) per milliliter of Whiskey using gallic acid calibration curve (R2=0.989). For this purpose, a calibration graph was constructed. The average value of triplicate measurements for each sample was used for calculation.

### 2.2 Total anti-oxidant capacity

The principle of determination of AOC in whiskey samples is based on Faraday's law where the mass of the analyte is determined by the amount of electricity spent on the reaction. The method was described by Lapin A. and Othman A.J. [2,3] and slightly modified as the following:

Exactly 1 mL of whiskey sample was transferred into the electrochemical cell containing 50 mL of buffer solution (0.2 M potassium bromide and 0.1 M sulfuric dioxide) with continuous stirring for 1 min. The electrolysis process begins when electric current generates Bromine anions at a constant current of 50 mA from the buffer solution.

The electrolysis begins at 40 millivolts, the initial, and the end value of the electrical titration were adjusted on 150 millivolts.

The initial and end values of electro-titration were set on 200 mV electrical current. Bromine anions were generated under 50 mA electrical current where all compounds with anti-Oxidants properties would react with the excessive bromine anions. The electrolysis process initiated at 40 mV. Total anti-oxidants capacity expressed as mg ascorbic acid equivalents per 100 mL of whiskey.

### 2.3 Statistical Analysis

Pearson's correlation coefficient, means, and standard deviations were performed using SPSS 25

## Ethics Statements

There is no funding for the present effort. There is no conflict of interest. The data is available in public domain.

## CRediT authorship contribution statement

**Ali J. Othman:** Conceptualization, Methodology, Software, Formal analysis, Writing – original draft. **Sarah G. Saker:** Software, Formal analysis, Writing – original draft.

## Declaration of Competing Interest

The authors declare that they have no known competing financial interests or personal relationships that could have appeared to influence the work reported in this paper.

## Data Availability

Dataset on the total phenolic contents and total anti-oxidants capacity in naturally distilled whiskey (Original data) (4tu.researchdata).

## Appendix Supplementary materials

Image, application 1
